# QRS complex configurations in 12-lead electrocardiograms of dogs with monomorphic ventricular tachycardia or complete bundle branch block

**DOI:** 10.3389/fvets.2025.1579951

**Published:** 2025-05-20

**Authors:** Manuela Perego, Alessandra Maffei, Damiano Cavallini, Roberto Santilli

**Affiliations:** 1 Clinica Veterinaria Malpensa Anicura, Samarate, Italy; 2 Department of Veterinary Medical Sciences, University of Bologna, Bologna, Italy; 3 Department of Clinical Sciences, College of Veterinary Medicine, Cornell University, Ithaca, NY, United States

**Keywords:** wide QRS complex, ventricular tachycardia, bundle branch block, 12-leads electrocardiogram, atrioventricular dissociation

## Abstract

**Introduction:**

The differentiation between ventricular tachycardias (VT) and supraventricular tachycardias (SVT) with bundle branch block (BBB) is clinically challenging. The aim of the study was to define by the 12-lead-electrocardiogram the QRS complex morphology in monomorphic VT (MVT) and in BBB.

**Methods:**

Twelve-lead-electrocardiograms were blindly retrospectively analyzed and categorized in four groups: sinus rhythm with left bundle branch block (SR-LBBB), sinus rhythm with right bundle branch block (SR-RBBB), MVT with RBBB configuration (MVT-RBBB), MVT with LBBB configuration (MVT-LBBB). Measurements were not normally distributed, and they were normalized by Box–Cox transformation. Repeated-measures linear mixed-effects models were constructed according to the 3 measurements performed.

**Results:**

A total of 103 12-lead-electrocardiograms were retrospectively analyzed: 18 SR-RBBB, 18 SR-LBBB, 33 MVT-RBBB, 34 MVT-LBBB. Limb leads concordance was found in 100% of SR-RBBB, 100% of SR-LBBB, 54.5% of MVT-RBBB, 70.6% of MVT-LBBB. Precordial leads discordance was present in 100% of SR-RBBB, 100% of SR-LBBB, 78.8% of MVT-RBBB, 88.2% of MVT-LBBB. The transition point was located at V1-V2 in 100% of SR-RBBB, 100% of SR-LBBB, 50.5% of MVT-RBBB and 71.5% of MVT-LBBB. Positive V1 with M shape morphology was detected in 100% of SR-RBBB and 12% of MVT-RBBB. The mean electrical axis on the frontal plane was -108.15° (−118.29 / −101.52) in RSSR-RBBB, 75.42° (71.78–80.46) in RSSR -LBBB, -93.46° (−102.75/−78.49) in MVT-RBBB and 82.27° (76.85–88.95) in MVT-LBBB.

**Discussion:**

In case of inability to identify signs of atrioventricular dissociation, the presence of standard limb leads discordance, precordial leads concordance or discordance with transition point other than V1-V2, left limb leads and left precordial leads discordance, aVR and V1 discordance and absence of M shape configuration of the QRS complex in lead V1 is likely to be MVT.

## Introduction

The diagnosis of wide QRS complex tachycardia (WCT) remains a common and often challenging clinical problem, as distinguishing between ventricular tachycardia (VT) and supraventricular tachycardia (SVT) with bundle branch block (BBB) is not always simple and intuitive, despite being fundamental for appropriate management and risk stratification (1–4). In patients presenting with regular WCT, it is crucial to rapidly differentiate potentially life-threatening VT from SVT with BBB, as the latter generally carries a more favorable prognosis (1–4).

In humans, VT and SVT with BBB account for the vast majority of WCT cases (80% and 15–20%, respectively) (3, 4). Other rare causes of WCT, such as pre-excited SVT, drug toxicity, electrolyte disorders (hyperkalemia), ventricular paced rhythms, and electrocardiographic artifacts, account for only a small minority of cases (1–5%) of WCT (3, 4). To the best of the author’s knowledge, no data are currently available regarding the differentiation among the various types of WCT in veterinary medicine.

Accurate and timely discrimination between VT and SVT with BBB through 12-lead electrocardiogram interpretation is essential for appropriate, high-quality, and cost-effective management of individuals presenting with WCT (5–22).

In human medicine, over the past 3 decades, several electrocardiographic criteria have been developed to help discern the underlying ventricular or supraventricular origin of WCT (5–22). Among these, the Brugada and Vereckei algorithms are the most commonly used (7, 9, 10, 13).

These criteria include the relationship between atrial and ventricular depolarizations, the duration of the QRS complex, the duration of the R-peak time, the initial R wave in lead aVR, the mean electrical axis of the QRS complex on the frontal plane, and the morphology of the QRS complex in the limb and precordial leads (5–22).

The aim of the study was to define the QRS complex configuration in monomorphic VT (MVT) and BBB to facilitate an accurate diagnosis of dogs presenting with regular monomorphic WCT.

## Materials and methods

The 12-lead electrocardiograms recorded at Clinica Veterinaria Malpensa between January 2019 and December 2023 were retrospectively and blindly analyzed by a third-year resident (AM) under the direct supervision of two board-certified cardiologists (RAS and MP). All included dogs underwent a 12-lead electrocardiogram, chest X-ray (right lateral and dorsoventral views), and standard transthoracic echocardiography. None of the enrolled dogs received any antiarrhythmic drugs.

The 12-lead electrocardiograms were recorded in non-sedated dogs gently restrained in right lateral recumbency. Standard limb leads (I, II, and III), unipolar augmented limb leads (aVR, aVL, and aVF), and precordial leads with V1 placed at the costochondral junction of the right first intercostal space were recorded. The sixth intercostal space was used for all the left-sided leads (V2 through V6), with lead V2 positioned adjacent to the sternum, V3 placed midway between V2 and V4, V4 at the level of the costochondral junction, and V5 and V6 sequentially positioned dorsal to V4 at a distance equal to that between V3 and V4, as previously described (23). For each wide QRS complex, three non-consecutive measurements were performed using a digital system (Easy ECG Smart - EB Neuro).

The electrocardiograms were categorized into four groups according to the following criteria: sinus rhythm with complete left bundle branch block (SR-LBBB), sinus rhythm with complete right bundle branch block (SR-RBBB), monomorphic ventricular tachycardia with an RBBB configuration (MVT-RBBB), and monomorphic ventricular tachycardia with an LBBB configuration (MVT-LBBB).

The inclusion criteria for the SR-RBBB group were as follows: normal P wave; normal and constant PQ interval; wide QRS complex (≥ 80 ms); deep S waves in leads I, II (>0.35 mV), III, and aVF and in left precordial leads; rightward deviation of the mean electrical axis of the QRS complex on the frontal plane (−100°/−120°); and Rr’, rR’, or RR’ morphology of the QRS complex in lead V1 (24).

The inclusion criteria for the SR-LBBB group were as follows: normal P wave; normal and constant PQ interval; wide QRS complex (≥ 80 ms); tall R waves in leads I, II (> 1 mV), III, and aVF and in left precordial leads; normal mean electrical axis of the QRS complex on the frontal plane (0°/+100°); and rS morphology of the QRS complex in lead V1 (24).

The inclusion criteria for the MVT-RBBB group were as follows: monomorphic WCT (heart rate ≥180 bpm and consistent beat-to-beat QRS complex morphology), QRS complex duration ≥80 ms, evidence of atrioventricular dissociation (P waves dissociated from the QRS complex, fusion beats, and capture beats), and R/S > 1 morphology of the QRS complex in lead V1 with a prominent R wave pattern and a positive QRS complex (4, 12, 25–27).

The inclusion criteria for the MVT-LBBB group were as follows: monomorphic WCT (heart rate ≥ 180 bpm and consistent beat-to-beat QRS complex morphology), QRS complex duration ≥80 ms, evidence of atrioventricular dissociation (P waves dissociated from the QRS complex, fusion beats, and capture beats), and R/S < 1 morphology of the QRS complex in lead V1with a slurred and delayed nadir of the S wave and a negative QRS complex (4, 12, 25–27).

For each electrocardiogram, the following electrocardiographic measurements were considered: heart rate, duration of the QRS complex (ms), mean electrical axis of the QRS complex on the frontal plane (°) using the previously reported formula [(arctan I_amp_, aVF_amp_) x 180/*π*] (28), duration of the R-peak time in precordial leads (ms), standard limb lead concordance or discordance, precordial lead concordance or discordance, transition zone of the QRS complex in precordial leads, configuration of the QRS complex in lead V1 (Rr’, rR’, or RR’, also referred to as an M-shape), left limb lead (I, II, III, and aVF) and left precordial lead (V2-V6) concordance, and right limb lead (aVR) and right precordial lead (V1) concordance.

The R-peak time is the duration it takes for the electrical wavefront to spread from the endocardial to the epicardial surface of the left ventricle. It is measured from the beginning of the QRS complex to the peak of the R wave. Left limb lead concordance occurs when all QRS complexes in lead I, II, III, and aVF are either predominantly positive (positive concordance) or negative (negative concordance). Left limb lead discordance refers to the QRS complexes having a downward direction in some of the leads (I, II, III, aVF). Precordial lead concordance means the QRS complexes have the same polarity, either predominantly positive (positive polarity) or negative (negative polarity) from V1 to V6. Precordial lead discordance occurs when the QRS complexes have a downward direction in some of the precordial leads. The transition zone in precordial leads is defined as the point where the QRS complex changes from being mostly negative to mostly positive, or vice versa. The transition point is determined by identifying in precordial leads where the R wave of the QRS complex becomes taller than the S wave or vice versa. Left limb lead and precordial lead concordance occurs when the QRS complex shows the same polarity in limb leads I, II, III, and aVF, as well as in precordial leads V2-V6. Left limb lead and precordial lead discordance refers to differing polarities of the QRS complex in the above-mentioned leads. Right limb lead and precordial lead concordance occurs when the QRS complex shows the same polarity in lead aVR and precordial lead V1, while right limb lead and precordial lead discordance indicates differing polarities of the QRS complex in the above-mentioned leads.

The normal distribution of the data was tested using the Shapiro–Wilk normality test (29). The measurements were not normally distributed, and they were normalized using Box–Cox transformation (30). Repeated-measures linear mixed-effects models were constructed (31) according to the electrocardiographic measurements obtained from three consecutive beats. The group was considered a fixed effect, while each subject was considered the experimental unit. After the analysis, the normal distribution of the data was re-evaluated for the resulting residuals. Means were reported as least square means, and pairwise multiple comparisons were performed using Tukey’s test as a post-hoc test when significance was detected (32). A nominal logistic model was used for categorical variables, applying the same discriminant criteria as previously described (33).

## Results

A total of 103 12-lead electrocardiographic tracings were retrospectively analyzed: 18 SR-RBBB, 18 SR-LBBB, 33 MVT-RBBB, and 34 MVT-LBBB.

The median age and weight of the dogs included in the study for the SR-RBBB group were 9 (1-14) years and 28.5 (3–53) kg, for the SR-LBBB group were 8 (0.5–12) years and 28.5 (12–57) kg, for the MVT-RBBB group were 9 (0.5–16) years and 28 (6–54) kg, and for the MVT-LBBB group were 8 (0.4–13) years and 25.5 (3–61) kg. No statistically significant differences were reported between the groups (*p* = 0.44 and 0.62, respectively).

Tables 1, 2 report the QRS complex morphology in the limb and precordial leads, as well as the electrocardiographic measurements for the QRS complex duration in lead II, mean electrical axis of the QRS complex on the frontal plane, and R-peak time in the precordial leads. In the SR-RBBB group, the median heart rate was 116 bpm (93–138); in the SR-LBBB group, 136 bpm (130–167); in the MVT-RBBB group, 260 bpm (229–290); and in the MVT-LBBB group, 279 bpm (233–312).

**Table 1:** Morphology of the QRS complex in the limb leads (I, II, III, aVR, aVL, and aVF) and precordial leads (V1, V2, V3, V4, V5, and V6).

LEAD	SR-RBBB	SR-LBBB	MVT-RBBB	MVT-LBBB
I	rS or rs (55.56%) qrS (16.68%) QS or qs (22.21%) qr. (5.55%)	qR or qr. (72.23%) R or r (27.77%)	qs or QS (35.36%) rS or rs (32.32%) R or r (15.15%) qr. or QR (12.12%) Rs or RS (5.05%)	r or R (45.06%) qr. or qR or Qr (26.48%) rs or rS (13,74%) QS or qs (8.84%) qRs (5,88%)
II	rS or rs (44.44%) qrS or qRS or qRS or qRS (44.44%) QS (11.12%)	qR or QR (66.67%) R (27.78%) qRs (5.55%)	QS (54.54%) rS or Rs or RS (33.34%) R (9.09%) qRS (3.03%)	R (54,90%) qR or QR or qr. (32.35%) Rs or rS (10.79%) QS (0,98%) qRs (0,98%)
III	rS or rs (44.44%) qRS or qrS (44.44%) QS (11.12%)	qR or QR (48.15%) R (46.30%) qRs (5.55%)	QS (54.52%) rS or Rs or RS (30.2%) R (9.07%) qRS or qRs (6.03%)	R (50.98%) qR or QR (33.34%) rS or Rs or RS (14.7%) QS (0.98%)
aVR	qR or qr. (66.66%) Rs (16.67%) R (16.67%)	rS (83.33%) QS (16.67%)	R (51.52%) qR or QR (33.33%) QS or qs (9,09%) Rs or rs (6.06%)	QS (55.88%) rS or Rs or RS (44.12%)
aVL	qR or qr. or Qr (50%) R or r (22.22%) rS or Rs or rs (22.22%) qs (5.56%)	QS or qs (75.92%) rS or RS or rs (24.08%)	R or r (55.55%) qR or Qr (20.2%) QS (15.16%) Rs or rS or Rs (9.09%)	QS or qs (61.77%) rS or Rs or RS (29.41%) qR or Qr (5.88%) qRs (2.94%)
aVF	rS or rs or RS (52.24%) qrS or qRS (36.64%) QS (11.12%)	qR or QR (61.12%) R (33.33%) qRs (5.55%)	QS (53.54%) rS or RS or Rs (32.32%) R (9.09%) qRS (5.05%)	R (51.97%) qR (32.35%) Rs or rS or RS or rs (11.76%) QS (2.94%) qRs (0.98%)
V1	Rr or rr (100%)	RS or rS (100%)	R or r (43.55%) Rr’ (12%) Rs or RS or rs (21.22%) qR or QR or qr. (20.2%) QRs or qRs (3.03%)	rS or RS (84.32%) QS (15.68%)
V2	RS or rS or rs (100%)	Rs or RS (42.43%) R (30.30%) qRs or qRS (18.18%) qR (9.09%)	RS or rS or Rs or rs (81.82%) R (12.12%) QS (6.06%)	Rs or rS or RS or rs (57.84%) R or r (35.3%) qR (5.88%) QS (0.98%)
V3	RS or rS or rs (90.91%) qRS (9.09%)	R (27.27%) RS (27.27%) qRs or qRS (27.27%) qR (18.19%)	RS or rS or Rs or rs (77.77%) R (15.16%) QS (7.07%)	R (48.06%) Rs or rS or RS (38.22%) qR (11.76%) QS (1.96%)
V4	RS or rS or rs (90.91%) qRS (9.09%)	qR (36.37%) R (27.27%) qRs or qRS (27.27%) RS (9.09%)	RS or rS or Rs or rs (75.76%) R (12.12%) QS or qs (12.12%)	R or r (51.98%) Rs or rS or rs or RS (29.4%) qR (9.80%) qRs (7.84%) QS (0.98%)
V5	RS or rS or rs (81.82%) qRS or qrs (18.18%)	qR or QR (54.55%) R (18.18%) qRs (18.18%) RS (9.09%)	RS or rS or Rs or rs (75.76%) QS (15.15%) R (9.09%)	R or r (49.04%) Rs or rS or rS or rs (26.46%) qR (17.64%) qRs (5.88%) QS (0.98%)
V6	RS or rS or rs (72.73%) qRS or qrS (18.18%) QS (9.09%)	qR or QR (45.46%) qRs or QRS (27.27%) R (18.18%) RS (9.09%)	rS or RS or Rs (75.76%) QS (15.15%) R (9.09%)	R or r (43.13%) qR (23.53%) Rs or RS or rs or rS (29.4%) qRs (2.94%) QS (0.98%)

The waves of the QRS complex are denoted using capital letters (Q, R, R’, and S) when the amplitude of the wave is ≥ 0.5 mV and lowercase letters (q, r, r’, and s) when the amplitude of the wave is < 0.5 mV. The Q wave is defined as the first negative wave of the QRS complex, the R wave as the first positive wave, the R’ wave as the second positive wave, and the S wave as the second negative wave.

**Table 2:** Duration of the QRS complex in lead II, mean electrical axis of the QRS complex on the frontal plane, and R-peak time in the precordial leads across the groups.

Electrocardiographic parameters	SR-RBBB	SR-LBBB	MVT-RBBB	MVT-LBBB
QRS duration in Lead II (ms)	98 (91–103.75)	104 (92–118)	87 (78–100)	96 (87.75–106)
QRS axis on the frontal plane (°)	−108.15 (−118.29/−101.52)	75.42 (71.78–80.46)	−93.46 (−102.75/−78.49)	82.27 (76.85–88.95)
R-peak time V1 (ms)	56 (50–66)	17 (13–19.5)	41 (34–52)	19 (14–24)
R-peak time V2 (ms)	21 (20.5–24-5)	50 (43–63)	27 (20–35)	36.5 (21–57.25)
R-peak time V3 (ms)	21 (17–24)	47 (42–54)	27 (18–35)	45.5 (24–61)
R-peak time V4 (ms)	20 (17–22)	51 (39–63)	27 (19–33)	48 (28.75–60)
R-peak time V5 (ms)	21 (13.5–25)	51 (46.5–63.5)	27 (18–35)	51 (34.75–60.25)
R-peak time V6 (ms)	22 (18.5–36)	51 (46–63)	22 (17.75–34)	52 (36.75–59.25)

The duration of the QRS complex was statistically longer in the SR-RBBB group compared to the MVT-RBBB group (*p* = 0.02), while no significant difference was observed in the SR-LBBB group compared to the MVT-LBBB group.

The R-peak time in V1 was statistically longer in the SR-RBBB group compared to the SR-LBBB group (*p* < 0.001) and in the MVT-RBBB group compared to the MVT-LBBB group (*p* < 0.001). However, there was no significant difference in the R-peak time between the SR-RBBB and MVT-RBBB groups or between the SR-LBBB and MVT-LBBB groups (*p* = 0.12 and *p* = 0.14, respectively).

The duration of the R-peak time in left precordial lead V5 was statistically longer in the SR-LBBB group compared to the SR-RBBB group (*p* < 0.001), in the MVT-LBBB group compared to the MVT-RBBB group (*p* < 0.001), and in the MVT-RBBB group compared to the SR-RBBB group (*p* < 0.001).

**Figure 1:**
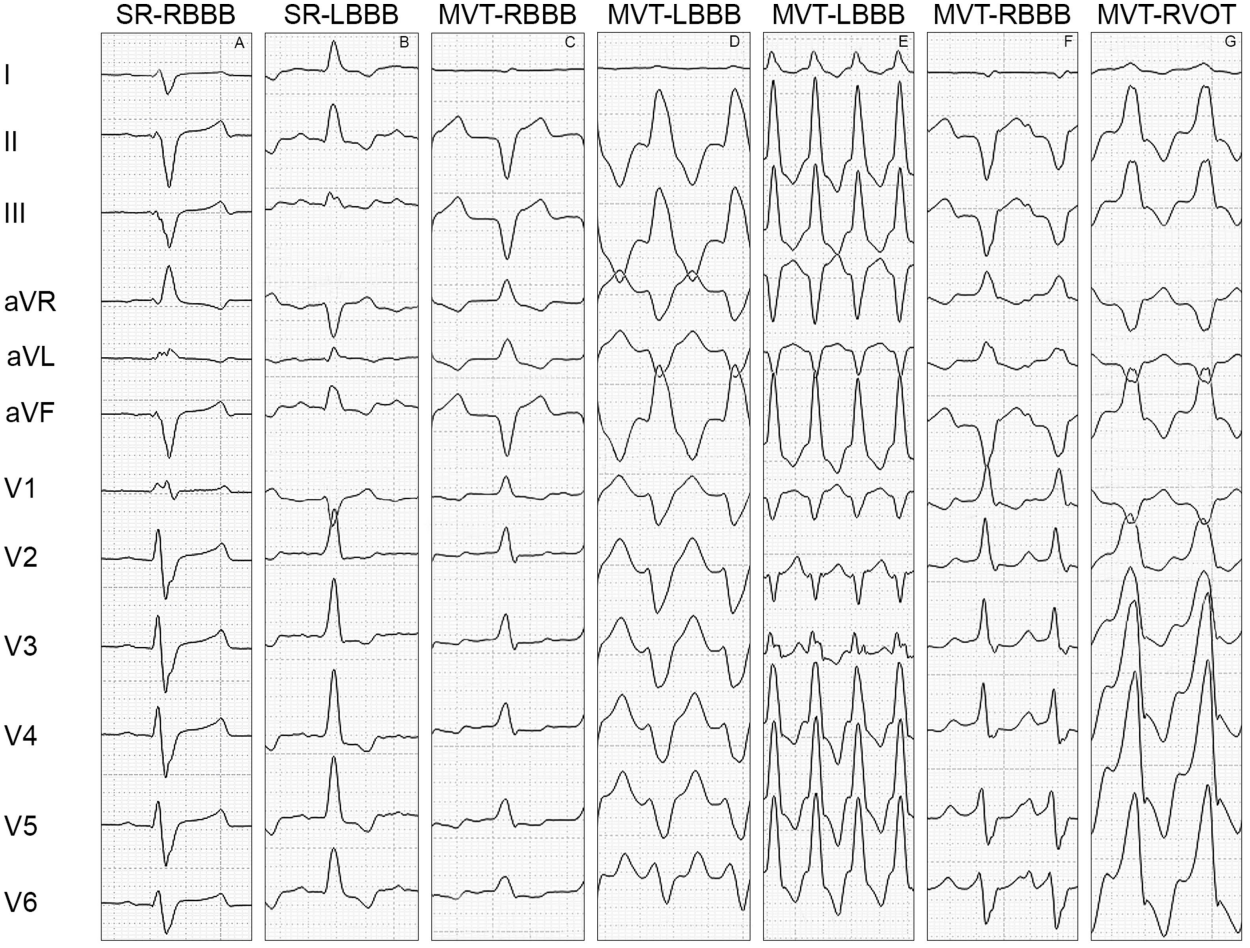
Morphology of the QRS complex in the limb leads (I, II, III, aVR, aVL, and aVF) and precordial leads (V1, V2, V3, V4, V5, and V6). **(A)** Illustrates the QRS complex morphology of a right bundle branch block, characterized by a positive polarity of the QRS complex in V1, negative concordance of the limb leads, discordance of the precordial leads with a transition point at V1-V2, negative concordance between the left limb leads (I, II, III, and aVF) and left precordial leads (V2-V6), positive concordance of the right limb lead (aVR) and right precordial lead (V1), and an M-shaped configuration of the QRS complex in V1. **(B)** Shows the QRS complex morphology of a left bundle branch block, characterized by a negative polarity of the QRS complex in V1, positive concordance of the limb leads, discordance of the precordial leads with a transition point at V1-V2, positive concordance between the left limb leads and left precordial leads (V2-V6), and negative concordance of the right limb lead and right precordial lead. **(C)** Displays the QRS complex morphology of monomorphic ventricular tachycardia with right bundle branch block, characterized by a positive polarity of the QRS complex in V1, discordance of the limb leads, positive concordance of the precordial leads, discordance between the left limb leads and left precordial leads, and positive concordance of the right limb lead and right precordial lead. **(D)** Shows the QRS complex morphology of monomorphic ventricular tachycardia with left bundle branch block, characterized by a negative polarity of the QRS complex in V1, positive concordance of the limb leads, negative concordance of the precordial leads, negative concordance between the left limb leads and left precordial leads, and discordance of the right limb lead and right precordial lead. **(E)** displays the QRS complex morphology of monomorphic ventricular tachycardia with left bundle branch block, characterized by a negative polarity of the QRS complex in V1, positive concordance of the limb leads, discordance of the precordial leads with a transition point at V2-V3, discordance between the left limb leads and left precordial leads, and positive concordance of the right limb lead and right precordial lead. **(F)** Illustrates the QRS complex morphology of monomorphic ventricular tachycardia with right bundle branch block, characterized by a positive polarity of the QRS complex in V1, negative concordance of the limb leads, discordance of the precordial leads with a transition point at V4-V5, discordance between the left limb leads and left precordial leads, and positive concordance of the right limb lead and right precordial lead. **(G)** Shows the QRS complex morphology of monomorphic ventricular tachycardia with left bundle branch block originating from the outflow tract of the right ventricle, characterized by a positive polarity of the QRS complex in V1, positive concordance of the limb leads, discordance of the precordial leads with a transition point at V1-V2, positive concordance between the left limb leads and left precordial leads (V2-V6), and negative concordance of the right limb lead and right precordial lead. SR-RBBB: sinus rhythm with right bundle branch block, SR-LBBB: sinus rhythm with left bundle branch block, MVT-RBBB: monomorphic ventricular tachycardia with right bundle branch block morphology, MVT-LBBB: monomorphic ventricular tachycardia with left bundle branch block morphology, MVT-RVOT: monomorphic ventricular tachycardia arising from the outflow tract of the right ventricle.

Left limb lead concordance was present in 100% of the SR-RBBB cases, with a negative QRS complex in leads I, II, III, and aVF (Figure 1A), and in 100% of the SR-LBBB cases (Figure 1B), with a positive QRS complex in leads I, II, III, and aVF.

Left limb lead concordance was present in 54.5% of the MVT-RBBB cases (Figure 1C) and in 70.6% of the MVT-LBBB cases (Figure 1D).

Precordial lead discordance was present in 100% of the SR-RBBB cases, with a positive QRS complex in V1 and a negative QRS complex in V2-V6 (Figure 1A), and in 100% of the SR-LBBB cases, with a negative QRS complex in V1 and a positive QRS complex in V2-V6 (Figure 1B). Precordial lead discordance was present in 78.8% of the MVT-RBBB cases and in 88.2% of the MVT-LBBB cases (Table 3).

**Table 3:** Electrocardiographic findings in the SR-RBBB, SR-LBBB, MVT-RBBB, and MVT-LBBB groups.

Electrocardiographic finding	SR-RBBB	SR-LBBB	MVT-RBBB	MVT-LBBB
Limb lead concordance	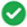 100%	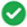 100%	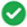 54.5% 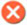 45.5%	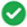 70.6% 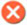 29.4%
Precordial lead discordance	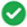 100%	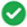 100%	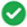 78.8% 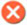 21.2%	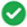 88.2% 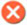 11.8%
Transition point V1-V2	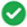 100%	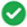 100%	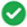 50.5% 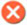 49.5%	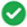 71.5% 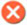 28.5%
Left limb lead and left precordial lead concordance	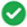 100%	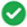 100%	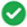 30% 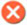 70%	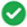 56.25% 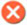 43.75%
Limb lead and right precordial lead discordance	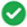 100%	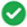 100%	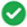 84.85% 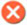 15.15%	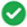 94.12% 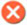 5.88%
M-shaped configuration of the QRS complex in lead V1	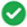 100%		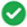 12% 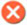 88%	
All the above findings present together	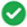 100%	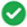 100%	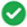 21.2% 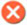 78.8%	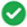 41.2% 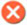 58.8%

The transition point was located at V1-V2 in 100% of the SR-RBBB cases (Figure 1A), 100% of the SR-LBBB cases (Figure 1B), 50.5% of the MVT-RBBB cases, and 71.5% of the MVT-LBBB cases. The transition point was located at V2-V3 in 16.2% of the MVT-RBBB cases and 8.8% of the MVT-LBBB cases (Figure 1E), at V3-V4 in 3% of the MVT-RBBB cases and 5% of the MVT-LBBB cases, at V4-V5 in 8.1% (Figure 1F) of the MVT-RBBB cases and 2.9% of the MVT-LBBB cases, and at V5-V6 in no cases (Table 3).

In lead V1, the QRS complex showed an Rr’, rR’, RR’, also known as M-shape configuration, in 100% of the SR-RBBB cases (Figure 1A) and 12% of the MVT-RBBB cases. No M-shape configuration was found in the SR-LBBB and MVT-LBBB groups.

The concordance between the left limb leads and left precordial leads (I, II, III, aVR, and V2-V6) was present in 100% of the SR-RBBB cases (negative QRS complex) (Figure 1A), 100% of the SR-LBBB cases (positive QRS complex) (Figure 1B), 30% of the MVT-RBBB cases, and 56.25% of the MVT-LBBB cases (Figures 1C–F).

The concordance between the right limb leads and right precordial leads (aVR and V1) was present in 100% of the SR-RBBB cases (positive QRS complex) (Figure 1A), 100% of the SR-LBBB cases (negative QRS complex) (Figure 1B), 84.85% of the MVT-RBBB cases, and 94.12% of the MVT-LBBB cases (Figures 1C–F).

In 41.2% of the MVT-LBBB cases, the QRS complex showed positive limb lead concordance and precordial lead discordance with a transition point at V1-V2, positive concordance between the left limb leads and left precordial leads, and negative concordance between the right limb lead and right precordial lead (Figure 1G). In 21.2% of the MVT-RBBB cases, the QRS complex showed negative limb lead concordance with precordial lead discordance and a transition point at V1-V2, negative concordance between the left limb leads and left precordial leads, and positive concordance between the right limb lead and right precordial lead.

## Discussion

To the best of the authors’ knowledge, this is the first study in canine electrocardiography to evaluate the QRS complex configuration in 12-lead electrocardiograms for MVT and sinus rhythm with a BBB (Table 3).

As reported in human literature, differentiating between MVT and SVT with BBB involves different steps (5–25). The first step is to identify the presence of atrioventricular dissociation (5–25). Atrial and ventricular activities are dissociated in MVT, and there are usually more QRS complexes than P waves. The presence of atrioventricular dissociation strongly favors the diagnosis of MVT. In human medicine, it has been proven that the sensitivity of atrioventricular dissociation is high (100%), while the specificity is low (20–50%) (9). This finding is correlated to the fact that it is difficult to identify P waves during WCT, as they are often hidden within the QRS complex or the T wave, as well as to the ventriculo-atrial conduction that is present in up to 50% of MVT (4, 12, 18). In dogs, the identification of atrial deflections is even more difficult compared to human beings, considering the faster rate of ventricular depolarization. In human studies, the likelihood of detecting atrioventricular dissociation is higher when using leads where the P wave is more prominent, such as the standard limb leads, including some of the inferior leads (specifically lead I, II, and III) (33, 34).

Other signs of atrioventricular dissociation include fusion beats and capture beats. Fusion beats result from the simultaneous ventricular activation of the wavefront from a supraventricular impulse and a premature ventricular ectopic impulse. The resulting QRS complex is neither identical to the premature ventricular ectopic beat nor to the QRS complex produced by the supraventricular impulse; instead, it appears as a hybrid between the two forms (16, 34, 35). Capture beats appear when a sinus impulse conducts through the atrioventricular node and captures the ventricle, producing a narrow QRS complex between the wide QRS complexes of the tachycardia. The RR interval between the last wide QRS complex and the narrow QRS complex is shorter compared to the RR interval of WCT.

Although the presence of signs of atrioventricular dissociation is highly suggestive of MVT, their absence does not exclude the diagnosis (34, 35). When atrioventricular dissociation is not present or easily identified, the QRS complex morphology must be evaluated across different leads to presume the underlying arrhythmia—whether MVT or SVT with BBB–based on the features of the depolarization vectors of the ventricles (polarity, amplitude, and duration) (12, 13, 34, 35).

The speed of cardiac depolarization varies according to the conduction properties of the tissue involved, being faster in the specialized conduction system and slower through the myocardial cells. In both BBB and MVT, this results in a widening of the QRS complex not only in its total duration but mainly at the expense of its initial part, the so-called ventricular activation or R-peak time, which is defined as the interval from the earliest onset of the QRS complex to the peak of the R wave (or R’ wave if present) and corresponds to the endo-epicardial activation time (24). In human medicine, a value greater than 140 ms in MVT-RBBB and a value greater than 160 ms in MVT-LBBB suggest MVT (5, 7, 35). In the present study, no statistically significant differences were found in the duration of the QRS complex in lead II and in the R-peak time in the precordial leads between the SR-RBBB and MVT-RBBB groups or between the SR-LBBB and MVT-LBBB groups.

Knowing the ventricular activation sequence in the case of complete right BBB or complete left BBB is crucial for differentiating MVT from SVT with BBB (35).

In complete right BBB, the first septal vector and the third septal vector have a normal direction and sequence of activation. The first septal vector typically proceeds from left to right, inferior to superior, and posterior to anterior, while the third septal vector moves from right to left, superior to inferior, and anterior to posterior. The second right ventricular vector is delayed with a normal sequence of activation: left to right, inferior to superior, and posterior to anterior (35). These vectorial features are correlated with the following electrocardiographic findings: negative limb lead concordance (lead I, II, III, and aVF), precordial lead discordance with a positive QRS complex in V1 and a transition point at V1-V2, negative left limb lead and left precordial lead concordance (lead I, II, III, aVF, and V2-V6), and positive right limb lead and right precordial lead concordance (aVR and V1) in 100% of the cases in the SR-RBBB group. The abnormalities in ventricular vectorial activation are also correlated with a shift to the right of the mean electrical axis of the QRS on the frontal plane (median value: −108.15) (35).

In complete left BBB, the first septal vector and the second septal vector have a normal direction and sequence of activation: left to right, inferior to superior, and posterior to anterior for the first vector, while left to right, inferior to superior, and posterior to anterior for the second vector. The third left ventricular vector is delayed with a normal sequence of activation: right to left, superior to inferior, and anterior to posterior (35). These vectorial features are correlated with the following electrocardiographic findings: positive limb lead concordance (lead I, II, III, and aVF), precordial lead discordance with a negative QRS complex in V1 and a transition point at V1-V2, positive left limb lead and left precordial lead concordance (lead I, II, III, aVF, and V2-V6), and negative right limb lead and right precordial lead concordance (aVR and V1) in 100% of the cases in the SR-LBBB group. The ventricular vectorial activation is also correlated with a normal mean electrical axis of the QRS on the frontal plane (median value: 75.42) (35).

Considering the above-mentioned QRS complex characteristics in both limb and precordial leads, WCT is likely to be MVT when these electrocardiographic findings are absent (35).

Based on the ventricular activation pattern in the frontal plane, standard limb lead discordance is less likely to occur in sinus rhythm with BBB. Monomorphic ventricular tachycardia with RBBB and MVT-LBBB showed standard limb lead discordance in 45.5 and 29.4% of the cases, respectively. The presence of standard limb discordance rules out BBB, even if standard limb lead concordance does not completely rule out MVT.

Based on the demonstrated vectors of the ventricular activation patterns seen in the horizontal (transverse) plane, precordial lead concordance is less likely to occur in sinus rhythm with BBB compared to MVT. Precordial lead concordance was found in 21.2% of the MVT-RBBB cases. Furthermore, precordial lead discordance with a transition point different from V1-V2 is unlikely to occur in sinus rhythm with BBB. The presence of precordial lead (V1-V6) discordance with a transition point different from V1-V2 was observed in 27.3% of the MVT-RBBB cases and 16.7% of the MVT-LBBB cases. The presence of precordial lead concordance or precordial lead discordance with a transition point different from V1-V2 rules out BBB, even if the presence of precordial lead discordance with a transition point between V1 and V2 does not completely rule out MVT.

These data are in agreement with human literature, which defines precordial concordance as a very specific pattern of MVT, with 100% specificity, although its sensitivity is low (26%) (7, 35).

The combined analysis of the vectorial activations in both frontal and horizontal planes revealed that the presence of left limb lead and left precordial lead (lead I, II, III, aVF, and V2-V6) discordance and right limb lead and right precordial lead discordance (aVR and V1) is unlikely to be correlated with BBB. Left limb lead and left precordial lead (I, II, III, aVF, and V2-V6) discordance was found in 70% of the MVT-RBBB cases and 43.75% of the MVT-LBBB cases.

Although positive left limb lead and left precordial lead (I, II, III, aVF, and V2-V6) concordance is typical of complete LBBB, this finding does not completely rule out MVT. In fact, it has previously been described that MVT originating from the right ventricle outflow tract caudal free wall typically shows positive limb lead concordance and precordial lead discordance with a transition point at V1-V2, positive left limb lead and left precordial lead concordance, and negative right limb lead and right precordial lead concordance (36). The inclusion of MVT originating from the outflow tract of the right ventricle in the present study can explain the 41.2% of the cases in the MVT-LBBB group presenting with QRS complex morphology similar to left BBB. To explain the 21.2% of the cases in the MVT-RBBB group presenting with QRS complex morphology similar to right BBB, further studies with electrophysiological mapping are needed to correctly localize MVT.

Right limb lead and right precordial lead discordance was present in 15.15% of the MVT-RBBB cases and 5.88% of the MVT-LBBB cases.

The configuration of the QRS complex in V1 is an important electrocardiographic finding to consider. In the case of complete right BBB, the delayed activation of the right ventricle produces a multi-peaked pattern with an initial R wave corresponding to the activation of the interventricular septum and a second R wave (R’ wave) corresponding to the slow activation of the right ventricle (35). This configuration can also be called M-shape in V1, and it was found in 100% of the SR-RBBB cases and 12% of the MVT-RBBB cases, suggesting that the absence of an M-shaped configuration in lead V1 rules out BBB, even if its presence does not rule out VT.

In humans, it has been established that the presence of a Q wave in lead aVR is correlated with the normal depolarization of the interventricular septum. This makes the presence of a positive initial R wave in lead aVR a finding with high specificity for MVT, with a specificity close to 99%. This finding is included in the algorithm used for the differentiation of MVT from BBB. The presence of an initial R wave in a wide QRS complex implies that the site of origin is from the ventricle, thus excluding BBB (3, 4, 10, 12, 20, 35). This electrocardiographic finding has also been described in a retrospective study in veterinary medicine, where the authors described the use of an algorithm that included the morphological criteria of the QRS complex in lead aVR to diagnose MVT. These criteria included the initiation of the QRS complex with a dominant R wave, the initiation of the QRS complex with a Q or R wave lasting longer than 40 ms, and the notching of the downstroke of a predominantly negative QRS complex (1). In the present study, contrary to previous reports, an R wave was detected in 33.32% of the SR-RBBB cases and 54.54% of the MVT-RBBB cases. Therefore, the presence of an R wave in lead aVR should not be considered a helpful electrocardiographic finding for diagnosing MVT. These findings are likely related to the different orientation and, consequently, the different vectorial direction of the interventricular septum on the frontal plane in dogs compared to humans.

The mean electrical axis on the frontal plane has been described in human medicine as a criterion for distinguishing MVT from BBB (35). The present study confirmed the previously reported values for SR-RBBB and SR-LBBB, but no statistically significant differences were found between SR-RBBB and MVT-RBBB or SR-LBBB and MVT-LBBB, even if in human medicine, it has been described that a right superior (north-west) axis deviation is suggestive of MVT. Specifically, a left superior (north-east) deviation in MVT-RBBB, or a right inferior (south-west) deviation in MVT-LBBB support the diagnosis (12) (Table 1).

A wide QRS complex with negative limb lead concordance and precordial lead discordance with positive V1 and a transition point at V1-V2, along with a mean electrical axis on the frontal plane deviated toward the right (−118.29°/−101.52°), is suggestive of right BBB. Conversely, a wide QRS complex with positive limb lead concordance and precordial lead discordance with negative V1 and a transition point at V1-V2, along with a normal mean electrical axis on the frontal plane (71.78° –80.46°), is suggestive of left BBB.

## Limitations

This study has some limitations: all dogs in the SR-RBBB and SR-LBBB groups had normal echocardiographic findings, while some dogs in the MVT-RBBB and MVT-LBBB groups presented with underlying structural heart disease, which could have influenced some electrocardiographic parameters such as the duration of the QRS complex and the mean electrical axis of the QRS complex on the frontal plane. The population of the dogs was grouped based on electrocardiographic abnormalities and not according to thoracic morphotype. Furthermore, in the MVT groups, despite the presence of atrioventricular dissociation, electrophysiological mapping was not performed to confirm the diagnosis and to localize the origin of the ventricular tachycardia.

## Conclusion

The use of a single electrocardiographic finding is unlikely to allow for accurate differentiation between MVT and BBB. However, wide QRS complexes presenting with standard limb lead discordance, precordial lead concordance or precordial lead discordance with a transition point other than V1-V2, left limb lead and left precordial lead discordance, right limb lead and right precordial lead discordance, and absence of an M-shaped configuration of the QRS complex in lead V1 are findings that strongly suggest MVT.

## Data Availability

The raw data supporting the conclusions of this article will be made available by the authors, without undue reservation.
