# Correction: QRS complex configurations in 12-lead electrocardiograms of dogs with monomorphic ventricular tachycardia or complete bundle branch block

**DOI:** 10.3389/fvets.2025.1758037

**Published:** 2026-04-13

**Authors:** Manuela Perego, Alessandra Maffei, Damiano Cavallini, Roberto Santilli

**Affiliations:** 1 Clinica Veterinaria Malpensa Anicura, Samarate, Italy; 2 Department of Veterinary Medical Sciences, University of Bologna, Bologna, Italy; 3 Department of Clinical Sciences, College of Veterinary Medicine, Cornell University, Ithaca, NY, United States

**Keywords:** wide QRS complex, ventricular tachycardia, bundle branch block, 12-leads electrocardiogram, atrioventricular dissociation

In the published article, there was a mistake in Table 3.

The content of the cell located in the **third column, sixth row**, was incorrect and it has been removed.

The corrected Table 3 appears below.

**Table 3:** Electrocardiographic findings in the SR-RBBB, SR-LBBB, MVT-RBBB, and MVT-LBBB groups.

**Electrocardiographic finding**	**SR-RBBB**	**SR-LBBB**	**MVT-RBBB**	**MVT-LBBB**
Limb lead concordance	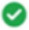 100%	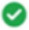 100%	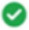 54.5% 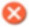 45.5%	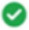 70.6% 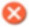 29.4%
Precordial lead discordance	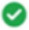 100%	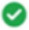 100%	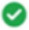 78.8% 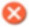 21.2%	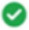 88.2% 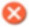 11.8%
Transition point V1–V2	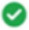 100%	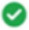 100%	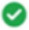 50.5% 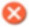 49.5%	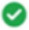 71.5% 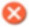 28.5%
Left limb lead and left precordial lead concordance	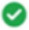 100%	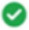 100%	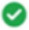 30% 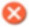 70%	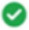 56.25% 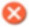 43.75%
Limb lead and right precordial lead discordance	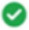 100%	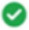 100%	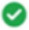 84.85% 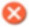 15.15%	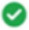 94.12% 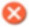 5.88%
M-shaped configuration of the QRS complex in lead V1	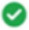 100%		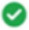 12% 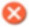 88%	
All the above findings present together	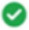 100%	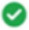 100%	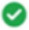 21.2% 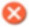 78.8%	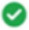 41.2% 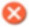 58.8%

The original version of this article has been updated.

